# Development of a new candidate vaccine against piglet diarrhea caused by *Escherichia coli*

**DOI:** 10.1515/biol-2022-0804

**Published:** 2023-12-31

**Authors:** Chongli Xu, Fengyang Fu, Yuhan She, Danni Yang, Kun Peng, Yimin Lin, ChongBo Xu

**Affiliations:** College of Medical Technology, Chongqing Medical and Pharmaceutical College, 82 Daxuecheng Road, Chongqing 401331, PR China; Chongqing Key Laboratory of Translational Research for Cancer Metastasis and Individualized Treatment, Chongqing University Cancer Hospital, Chongqing 400030, PR China; School of Biology and Agriculture, Shaoguan University, Shaoguan 512005, PR China

**Keywords:** K88ac-K99-3ST_1_-LT_B_, gene fusion, recombinant strain, minimum immunization dose, vaccine

## Abstract

Enterotoxigenic *Escherichia coli* (ETEC) is an important type of pathogenic bacteria that causes diarrhea in humans and young livestock. The pathogen has a high morbidity and mortality rate, resulting in significant economic losses in the pig industry. To effectively prevent piglet diarrhea, we developed a new tetravalent genetically engineered vaccine that specifically targets ETEC. To eliminate the natural toxin activity of ST_1_ enterotoxin and enhance the preventive effect of the vaccine, the mutated *ST*_*1*_, *K88ac*, *K99*, and *LT*_*B*_ genes were amplified by PCR and site-specific mutation techniques. The recombinant strain BL21(DE3)(pXKK3SL) was constructed and achieved high expression. Animal experiments showed that the inactivated vaccine had eliminated the natural toxin activity of ST_1_. The immune protection test demonstrated that the inclusion body and inactivated vaccine exhibited a positive immune effect. The protection rates of the inclusion body group and inactivated vaccine group were 96 and 98%, respectively, when challenged with 1 minimum lethal dose, indicating that the constructed K88ac-K99-3ST_1_-LT_B_ vaccine achieved a strong immune effect. Additionally, the minimum immune doses for mice and pregnant sows were determined to be 0.2 and 2 mL, respectively. This study suggests that the novel K88ac-K99-3ST_1_-LT_B_ vaccine has a wide immune spectrum and can prevent diarrhea caused by ETEC through enterotoxin and fimbrial pathways. The aforementioned research demonstrates that the K88ac-K99-3ST_1_-LT_B_ vaccine offers a new genetically engineered vaccine that shows potential for preventing diarrhea in newborn piglets.

## 1 Introduction

Enterotoxigenic *Escherichia coli* (ETEC) is a significant pathogen that causes diarrhea in young animals, such as piglets, calves, and lambs. ETEC is also among the main pathogens responsible for the high incidence and mortality of bacterial diarrhea in humans and animals [1,2,3,4]. When infected with ETEC, individuals often experience severe watery diarrhea and rapid dehydration, which greatly impacts human health and the sustainable development of aquaculture [5,6]. ETEC-induced diarrhea has become a major contributor to infant diarrhea in developing countries, as well as a cause of diarrhea in children, adults, and tourists [7,8,9,10]. Patients and carriers are the primary sources of infection, which can contaminate the surrounding environment and spread rapidly. Many outbreaks have been linked to water, food, milk, and beverages. People are generally susceptible to ETEC, and many tourists experience severe diarrhea due to food and water contamination by ETEC [11,12]. ETEC belongs to the Enterobacteriaceae genus and is a gram-negative bacterium. The optimal growth temperature for ETEC is 37°C, and the optimal pH value is 7.2–7.4 [13,14]. The antigens within ETEC are complex and primarily include cell O antigen, flagellar H antigen, and capsule K antigen, and the pathogen has different serotypes in different regions. ETEC produces toxic factors, such as adhesin, enterotoxin, endotoxin, and hemolysin. Enterotoxin and adhesin play important roles in pathogenesis and immunology. Adhesion antigens mainly include F4 (K88), F5 (K99), F18, and F41 [15,16]. F5 (K99), F6 (987P), and F41 were almost exclusively found in pigs less than 1 week old. F4 (K88) can cause diarrhea in piglets after they are weaned, and F18 was only discovered in weaned piglets. Based on its antigenicity, F18 was divided into two antigen variants, F18ab and F18ac. The F18ac strain was linked to postweaning diarrhea, while F18ab was associated with edema disease. Infection with F4 or f18 usually leads to sudden death or reduced feed intake and watery diarrhea in one or several pigs, most often in the first week after weaning. Diarrhea can be much more severe after weaning than immediately after birth and usually only results in weight loss.

ETEC fimbriae bind to various receptors in the intestinal mucosal epithelium and attach to intestinal epithelial cells, preventing rapid excretion caused by constant peristalsis in the animal digestive tract. This process creates conditions favorable for a significant increase in bacterial growth and multiplication, leading to the production of enterotoxins that cause disease. Adhesin is a protein-based antigen with strong immunogenicity. Immunizing animals with bacteria or purified adhesin antigen can efficiently produce the corresponding antibodies. ETEC attaches to the epithelial cells of the host’s intestinal mucosa with the assistance of adhesin and produces a large amount of enterotoxin, resulting in pathological changes in the intestinal mucosal epithelial cells and causing diarrhea in piglets [17,18,19]. Enterotoxins can generally be classified as heat-stable enterotoxin (ST) and heat-labile enterotoxin (LT). Strains can produce ST or LT alone or both toxins. Enterotoxin plays a crucial role in ETEC diarrhea, so studying its pathogenesis and immunogenicity is very important for preventing and controlling diarrheal diseases caused by ETEC. Research results have shown that 20–30% of the ETEC strains causing piglet diarrhea were LT^+^/ST^−^. Thirty to forty percent of ETEC strains were LT^+^/ST^+^, while LT^−^/ST^+^ strains accounted for nearly 50% [20]. Therefore, compared to LT, ST plays a vital role in diarrheal diseases in young livestock caused by ETEC. ST is further divided into ST_1_ and ST_2_. ST_1_ includes ST_1a_ (porcine-type, STp) and ST_1b_ (human-type STh). The genes encoding ST enterotoxin are located on the plasmid, and ST does not possess immunogenicity. LT consists of a single A subunit (∼28 kDa) and five B subunits (∼11.5 kDa each). LT and its B subunit exhibit good immunogenicity [21,22,23,24].

The treatment for ETEC disease primarily relies on drugs and vaccines. However, ETEC is often resistant to multiple antibiotics, so vaccination with vaccines, such as F4 or F18, can effectively prevent piglet diarrhea. Additionally, vaccinating sows can prevent colibacillosis in newborn piglets, but it does not completely prevent diarrhea after weaning. More importantly, vaccination remains the most effective method of disease control due to the emergence of drug residues and bacterial resistance, which has resulted from the excessive use of antibiotics in recent years. Commercial or experimental vaccines used to prevent ETEC include inactivated whole vaccines, subunit vaccines, and genetically engineered vaccines. The main virulence factors of ETEC, adhesin and enterotoxin, are crucial factors that cause diarrhea in piglets. Although vaccines are available that provide some preventive protection, all vaccines for ETEC exhibit certain limitations. Some vaccines do not address the toxicity caused by the major enterotoxin ST, while others only target a single adhesin or enterotoxin. The immune effect of the vaccines is not optimal. Therefore, developing a new broad-spectrum vaccine to prevent diarrhea in newborn piglets is very important. Seo et al. constructed BSA-STa_A14T_ and 3xSTa_N12S_-mnLT_R192G/L211A_ and immunized pigs. The piglets were better protected by the induced anti-STa antibodies [25]. Feng and Guan constructed a recombinant strain expressing LTA-STa_A13Q_-STb-LTA2-LTB-STa_A13Q_-STb. After immunization with the bacteria, the serum IgG and fecal sIgA responded against all ETEC enterotoxins and induced F41 antibody in mice. Moreover, there were higher levels of IL-4 than IFN-ϒ, suggesting a T-cell (Th) response [26]. Lu et al. developed the multiepitope fusion antigen (MEFA). The fimbria toxin MEFA generated induced neutralizing antibodies against ETEC fimbriae and all four ETEC toxins. Because MEFA-based vaccines do not carry somatic antigens, they cause fewer side effects [27]. However, the immune response is not ideal, as these vaccines do not address the immunity of ST_1_, which is the most crucial toxin factor that causes diarrhea in piglets. If this important factor is not effectively resolved, the vaccine will not be effective in preventing the disease and will result in significant economic losses. We created a genetically engineered strain, K88ac-K99-ST_1_-LT_B_, through gene mutation and gene fusion technology; this strain targets the main virulence factors of adhesion K88ac and K99, as well as enterotoxin ST_1_ and LT_B_ of ETEC. This recombinant strain not only rendered ST_1_ inactive as a natural enterotoxin but also endowed it with immunogenicity. The prepared K88ac-K99-3ST_1_-LT_B_ tetravalent genetically engineered inactivated vaccine can prevent diarrhea caused by ETEC through enterotoxin and fimbriae. The results demonstrated that the K88ac-K99-3ST_1_-LT_B_ vaccine was an ideal vaccine for preventing piglet diarrhea, effectively controlling the occurrence of diarrhea in newborn piglets and yielding significant economic and social benefits.

## 2 Materials and methods

### 2.1 Construction of a candidate strain for a tetravalent genetically engineered vaccine

Corresponding primers were designed according to the reported *ST*_*1*_, *LT*_*B*_, *K88ac*, and *K99* gene sequences [10,28]. Three pairs of ST_1_ site-specific mutation primers were designed and synthesized. The upstream primers the P1, P2, and P3 contained *HindIII*, *NdeI*, and *EcoRI* cleavage sites (italics) and protective bases, respectively, while the downstream primers P4, P5, and P6 contained *NdeI*, *EcoRI*, and *BamHI* cleavage sites (italics) and protective bases, respectively [29]. Two cysteines were mutated to serines (TGT→AGT) at the 3′ end of the *ST*_*1*_ gene using three pairs of mutant primers. Three *ST*_*1*_ mutant genes were amplified from the *E. coli* C83902 plasmid by a PCR site-specific mutation technique. The *LT*_*B*_ gene was amplified from the C83902 plasmid by PCR amplification using the P7 and P8 primers. The P7 and P8 primers contained *Bam*H I and *Not* I cleavage sites. Using P9 and P10 primers, the *K88ac* gene was amplified from the template C83902 plasmid. The P9 and P10 primers contained *Bgl* Ⅱ and *Nco* I cleavage sites [29]. The *K99* gene was amplified from the C83539 plasmid using P11 and P12 primers. The P11 and P12 primers contained *Nco* I and *Hin*d III restriction endonuclease sites [30]. Using DNA ligase, the amplified *K88ac*, *K99*, *ST*_*1*_, and *LT*_*B*_ genes were concatenated into the K88ac-K99-3ST_1_-LT_B_ fragment. The above primers are shown in Table 1. The PCR system included the following components: 1 µL template, 2 µL 5 mmol/L dNTP, 2 µL 0.1 mol/L primer, 5 µL 10× buffer, 1 µL 3 U/µL Taq DNA polymerase, and 39 µL ddH_2_O. The PCR amplification procedures were performed as follows: predenaturation at 95°C for 5 min, denaturation at 94°C for 30 s, annealing at 50°C for 30 s, extension at 72°C for 1 min, and 30 cycles. After PCR amplification, the product and pET-28a were digested with *BglII* and *Not*I, respectively. The mixture was incubated with T_4_ DNA ligase overnight at 16°C. After the recombinant plasmid was transformed into BL21(DE3), the recombinant bacteria were coated on Kan/LB plates and cultured overnight at 37°C. After overnight cultivation, the recombinant plasmid pXKK3SL was extracted for restriction digestion identification and nucleotide sequence analysis.

**Table 1:** Primer sequence

Primer	Primer sequence
P1	5′-CCC ***AAGCTT*** AACAACACATTTTACTGC-3′
P2	5′-GGAATTC ***CATATG*** ATAACTTCCAGCACTGGC-3′
P3	5′-GGAATTC ***CATATG*** AACAACACATTTTACTGC-3′
P4	5′-CCG ***GAATTC*** ATAACTTCCAGCACTGGC–3′
P5	5′-CCG ***GAATTC*** AACAACACATTTTACTGC-3′
P6	5′-CGC ***GGATCC*** ATAACTTCCAGCACTGGC-3′
P7	5′-GGA ***AGATCT*** CATTTACTGACTATGAAGAA-3′
P8	5′-CATG ***CCATGG*** GAGAATATCATTTCTTGATAG-3′
P9	5′-CATG ***CCATGG*** A GAT CTT GGG CAG CCT CCT-3′
P10	5′-CCC ***AAGCTT*** AT ATA AGT GAC TAA GAA-3′
P11	5′-CGC ***GGATCC*** CCAGACTATTACAGAACTA-3′
P12	5′-ATAAGAAT ***GCGGCCGC*** AAGCTTGCCCCTCCAGCCTAG C-3′

### 2.2 Induced expression of strain, SDS‒PAGE detection, and ELISA analysis of fusion protein

According to a method described by Sambrook et al. [31], the recombinant strain BL21(DE3)(pXKK3SL) was spread on a Kan/LB plate and incubated overnight at 37°C. Then, a single colony was selected and inoculated into 5 mL of liquid LB medium containing 30 μg/mL kanamycin. The culture was then incubated overnight in a shaking incubator at 37°C with a speed of 170 rpm. Next, the culture was inoculated into a culture bottle containing 250 mL of LB medium at a ratio of 1%. IPTG was added to the culture bottle to a final concentration of 1 mmol/L. The culture was then incubated at 37°C at a speed of 170 rpm for 4 h to reach the logarithmic growth phase (OD_600_ = 0.4–0.6). The recombinant strain BL21(DE3)(pXKK3SL) was induced and cultured, and 1 mL of the culture was centrifuged at 12,000 rpm. The supernatant was discarded, and the bacterial pellet was collected. The precipitate was resuspended in 0.5 mL of 50 mmol/mL Tris-Cl (pH 7.4) and centrifuged at 0°C at 12,000 rpm for 30 s. The supernatant was discarded, and the precipitate was resuspended in 25 μL of water. Once the bacteria were dispersed, 25 μL of 2× SDS gel loading buffer was immediately added. After being oscillated for 20 s, the supernatant was placed in a boiling water bath for 3–5 min and then centrifuged at 12,000 rpm for 10 min. Twenty-five microlitres of the supernatant were taken for SDS‒PAGE electrophoresis. BL21(DE3)(pXKK3SL), the negative control BL21(DE3)(pET-28a), the positive control ST^+^/HB101(pSLM004), and LT^+^/DH5α(pEWD299) strong strains were split by ultrasonic waves. The lysate was detected by ELISA.

### 2.3 Fusion protein antigen preparation

The 50 mL culture induced by IPTG for 4 h was centrifuged, and the bacteria were collected and resuspended in 5 mL of TE (50 mmol/L Tris-Cl, 2 mmol/L EDTA). The bacteria were then added to lysozyme at a final concentration of 100 μg/mL and 5 mL of 1% Triton X-100 and incubated at 30℃ for 15 min. The lysate was treated with an ultrasonic wave two times for 10 s each and then centrifuged at 12,000 rpm for 15 min. After the precipitate was collected and diluted 10 times, a final concentration of 10% aluminum hydroxide glue was added and mixed as an immune antigen. Additionally, formaldehyde was added to the culture medium of the engineered bacteria at a final concentration of 0.4% to inactivate the bacteria. After 10% aluminum hydroxide glue was added, the mixture was used as an immunological antigen [29].

### 2.4 Safety test for the recombinant strain BL21(DE3)(pXKK3SL) and minimum lethal dose (MLD)

#### 2.4.1 Determination of challenge strain

To determine whether the K88ac-K99-3ST_1_-LT_B_ fusion protein had eliminated the natural toxin activity of ST_1_ enterotoxin, 40 mice weighing 18–22 g were randomly divided into eight groups of five mice. Groups 1–4 were injected with the recombinant strain BL21(DE3)(pXKK3SL) intraperitoneally, and groups 5–8 received the strain through oral inoculation. The clinical response of the test mice was observed daily, and autopsies were performed after continuous observation for three weeks. Fifty mice weighing 18–22 g were randomly divided into five groups of ten mice. Groups 1–4 were separately challenged using different doses of strains (0.5 × 10^9^ CFU, 1.0 × 10^9^ CFU, 1.5 × 10^9^ CFU, 2.0 × 10^9^ CFU). Group 5 was used as the control group. After 3 days of observation, the MLD was determined based on the death of the mice. After birth, 15 piglets that did not receive colostrum were selected and divided into 5 groups, each consisting of 3 piglets. Groups 1–4 were exposed to different doses of strains (1.0 × 10^11^ CFU, 1.5 × 10^11^ CFU, 2.0 × 10^11^ CFU, 2.5 × 10^11^ CFU). Group 5 served as the control group.

#### 2.4.2 Immune protection test

Three hundred mice weighing 18–22 g were randomly divided into six groups of 50 mice each. Groups 1 and 2 were injected intraperitoneally with inclusion bodies, while groups 3 and 4 were injected intraperitoneally with inactivated vaccines of genetically engineered strains. The animals received two injections at a 14-day interval at a dose of 0.2 mL per animal. Fourteen days after the second immunization, the mice were challenged with 1 MLD and 2 MLD of virulent strains C83902 and C83539. The death of the mice was then observed daily. Groups 5 and 6, injected intraperitoneally with the K88-LT_B_ bivalent vaccine, were used as the negative control.

#### 2.4.3 Determination of the minimum immune dose in mice

Ninety mice were divided into six groups. Groups 1, 3, and 5 (20 each group) were immunized with different doses (0.15, 0.2, 0.25 mL/mouse). All mice were immunized twice with a 14-day interval. As the control group, group 2, group 4, and group 6 (10 each group) were only injected with saline. After the second immunization was completed, all mice were injected with 1 MLD C83902 and C83539 virulent strains and observed for 7 days. The immune protection rates of different groups were recorded in detail.

Eight pregnant sows were divided into four groups, with two sows in each group. Group 1 and group 3 were immunized twice through the neck muscles, 30–35 and 15–20 days before delivery. The immunization doses for group 1 and group 3 were 2 and 4 mL/sow of vaccines, respectively. As the control group, group 2 and group 4 were only injected with saline. Newborn piglets were challenged with 1 MLD C83902 and C83539 virulent strains on the first day after colostrum was administered. The clinical reaction was observed daily after the challenge and the immune protection rates of all groups were recorded in detail.


**Ethical approval:** The research related to animal use has been complied with all the relevant national regulations and institutional policies for the care and use of animals and has been approved by the Institutional Animal Care and Use Committee and Ethics Committee of Chongqing Medical and Pharmaceutical College.

## 3 Results

### 3.1 Induced expression of the recombinant strain BL21(DE3)(pXKK3SL)

**Figure 1:**
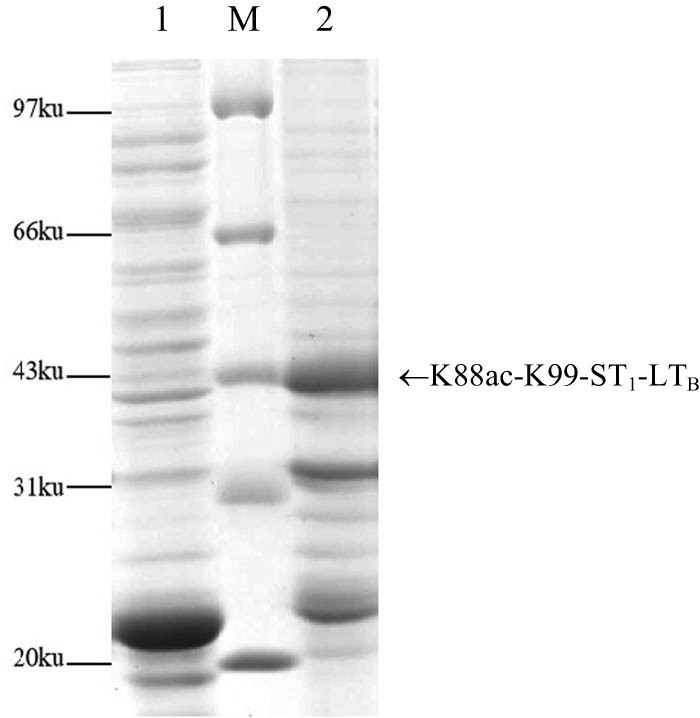
SDS‒PAGE analysis of BL21(DE3)(pXKK3S) expression. M: low molecular protein marker; (1) total cell lysate of BL21(DE3)(pET-28a) and (2) total cell lysate of BL21(DE3)(pXKK3S).

The mutant 180-bp ST1 gene (Cys → Ser) was cloned using the C83902 plasmid as a template through a site-specific mutation technique. The 330-bp K88ac and 500-bp LTB gene fragments were amplified by PCR using the C83902 plasmid as a template. The C83539 plasmid of *E. coli* was extracted, and the 393-bp *K99* gene fragment was amplified by PCR. The 1299-bp *K88ac-K99-3ST*_*1*_ *-LT*_*B*_ fusion gene fragment was constructed using T4 DNA ligase. After the fusion gene *K88ac-K99-3ST*_*1*_ *-LT*_*B*_ was cut by a restriction enzyme, the gene was cloned and inserted into pET-28a to generate the recombinant expression plasmid pXKK3SL. After enzyme digestion and sequence analysis were performed, the plasmid pXKK3SL was transformed into *E. coli* BL21(DE3). The recombinant strain BL21(DE3)(pXKK3SL) was induced by IPTG for 4 h at 37°C. SDS‒PAGE analysis showed that the fusion protein accounted for 35.72% of the total protein content of the bacteria (Figure 1). Therefore, efficient expression of the K88ac-K99-3ST_1_-LT_B_ fusion gene was achieved. ELISA showed that the K88ac-K99-3ST_1_-LT_B_ fusion protein can bind to ST_1_ monoclonal antibody, LT_B_, K88ac and K99 antibody.

### 3.2 Safety test for BL21(DE3)(pXKK3SL) and challenge protection test

To confirm whether the K88ac-K99-3ST_1_-LT_B_ fusion protein had eliminated the natural toxin activity of ST1, mice were inoculated with the BL21(DE3)(pXKK3SL) strain through intraperitoneal injection and orally. After 3 weeks of observation, all mice survived, and no pathological changes were found during autopsy. This result indicated that the recombinant strain had eliminated the natural toxin activity of ST_1_ and was safe and nonpathogenic to mice. The experimental results showed that the MLD for mice was 1.0 × 10^9^ CFU, and for piglets, the dose was 1.5 × 10^11^ CFU. Mice were immunized with inclusion bodies and inactivated vaccines separately and were effectively protected when challenged by the virulent strains C83902 and C83539. With 1 MLD challenge to mice, the protection rate of the inclusion body immunization group was 96% (48/50), and the protection rate of the inactivated vaccine immunization group was 98% (49/50). With 2 MLD challenges to mice, the protection rate of the inclusion body immunization group was 94% (47/50), and the protection rate of the inactivated vaccine immunization group was 96% (48/50). The protection rates in the control group were 88% (44/50) and 82% (41/50) (Table 2). The immune protection rate for the 1 and 2 MLD challenge doses exceeded 90%. However, there was no significant difference in immune effect between the inclusion body group and the inactivated vaccine group. There was little difference in the immune protection effect.

**Table 2:** Results of the mouse challenge protection test

Challenge dose (MLD)	Survival/immune number			Immunity way	Immunity frequency	Immunity dose (mL)	Immunization interval (days)
	Inclusion body group	Inactivated vaccine group	Control group				
1	48/50	49/50	44/50	Intraperitoneal injection	Twice	0.2	14
2	47/50	48/50	41/50	Intraperitoneal injection	Twice	0.2	14

### 3.3 Determination of the minimum immune dose

The protection rates for group 1, group 3, and group 5 of mice were 75% (15/20), 85% (17/20), and 95% (19/20), respectively, after being challenged with a 1 MLD dose. All mice in the control group died. The results indicate that the minimum immune dose of the vaccine for mice was 0.2 mL/mouse. The protection rates for group 1 and group 3 of piglets were 86.9% (20/23) and 92.3% (24/26), respectively, after being challenged with a 1 MLD dose. Only four piglets experienced very mild diarrhea. However, all piglets in the control group died. The results indicate that the minimum immune dose of the inactivated vaccine for pregnant sows was 2 mL/pig (Tables 3 and 4). Compared to pigs immunized with a 2 mL dose, pigs immunized with a 4 mL dose achieved a higher immune protection rate; however, the difference was not significant. Therefore, considering the cost factor, the minimum immune dose was determined to be 2 mL.

**Table 3:** Results of minimum immunization dose of mice

Group	Reagent	Immunization dose (mL)	Challenge dose (MLD)	Amount	Number of survivor	Protection rate (%)
Group 1	Inactivated vaccine	0.15	1	20	15	75
Group 2	Normal saline	0.15	1	10	0	0
Group 3	Inactivated vaccine	0.20	1	20	17	85
Group 4	Normal saline	0.20	1	10	0	0
Group 5	Inactivated vaccine	0.25	1	20	19	95
Group 6	Normal saline	0.25	1	10	0	0

**Table 4:** Results of minimum immunization dose of pregnant sows

Group	Reagent	Immunization dose (mL)	Challenge dose (MLD)	Amount	Number of survivor	Protection rate (%)
Group 1	Inactivated vaccine	2.0	1	23	20	86.9
Group 2	Normal saline	2.0	1	17	0	0
Group 3	Inactivated vaccine	4.0	1	26	24	92.3
Group 4	Normal saline	4.0	1	20	0	0

## 4 Discussion

Diarrhea is the primary cause of piglet mortality and results in significant economic losses in the pig industry. Viruses, bacteria, and improper diet can contribute to diarrhea in piglets. For instance, soybean can easily lead to weaning diarrhea because trypsin inhibitors or antigens cause a local immune response [31]. Currently, ETEC is the primary pathogen responsible for piglet diarrhea. This condition has consistently impeded progress in the pig industry. The incidence of ETEC can range from 20 to 40%, with mortality rates reaching 30–50% [32,33,34]. Even if pigs are cured with antibiotics, their production performance indicators are greatly reduced, and they completely lose their economic value for feeding, resulting in substantial economic losses for the pig industry. The virulence factors of ETEC include adhesin and enterotoxin. The main adhesins are K88 and K99. With the assistance of these adhesins, ETEC attaches to the epithelial cells of the host’s intestinal mucosa and produces a significant amount of enterotoxin, which causes pathological changes in the intestinal mucosal epithelial cells and leads to piglet diarrhea. The main adhesins of ETEC derived from pigs are K88ac and K99, which indirectly cause diarrhea in piglets. Enterotoxins, such as heat-ST and heat-LT, directly cause diarrhea in piglets. ST is divided into ST_1_ and ST_2_ based on antigenicity and host differences. ST_1_ is a small peptide consisting of 18 or 19 amino acids that remain active even when heated at 100°C for 30 min. ST_1_ is highly toxic but lacks immunogenicity. LT is composed of an A subunit and a B subunit and both LT and its B subunit exhibit good immunogenicity [35,36].

Diarrhea in piglets caused by ETEC has always been a main focus and challenge in research. The difficulty lies in finding a method to eliminate the biological toxicity of ST_1_ and generate immunogenic properties. ST_1_ enterotoxin contains six cysteine residues, which can form three pairs of disulfide bonds within the chain. These disulfide bonds are crucial for the toxicity of ST_1_. If these bonds are broken, ST_1_ can lose its biological toxicity. On the other hand, the LT_B_ subunit exhibits strong immunogenic properties. Previous studies have shown that LT_B_ can be used as a carrier protein for ST. As a result, ST can acquire immunogenicity and stimulate an immune response against ST and LT. Therefore, the fusion of *LT*_*B*_ and *ST* genes has become the focus of research for a potential polyvalent vaccine strain. Some researchers constructed a recombinant strain that contained the *ST*_*1*_ *–LT*_*B*_ fusion gene and inserted a 21 bp linker gene between the two genes. The results showed that the ST_1_–LT_B_ fusion protein lost its ST_1_ enterotoxin activity and exhibited good immunogenicity [37]. Other researchers also constructed the *ST*_*1*_ *–LT*_*B*_ fusion gene, but the expression product still showed ST_1_ enterotoxin activity [38]. Additionally, the *pro-ST*_*1*_ gene, which encodes the ST_1_ precursor protein, was fused to the 3′-terminus of the *LT*_*B*_ gene. The LT_B_-pro-ST_1_ fusion protein exhibited good immunogenicity and lost its ST_1_ enterotoxin activity [39]. Numerous studies have shown that different serotypes of *E. coli*, which cause piglet diarrhea, have different colonization factors. Among them, K88ac and K99 are the dominant fimbriae [40,41].

Currently, no universal protective ETEC vaccine is available for piglet diarrhea, although adhesion-based vaccines provide some level of protection. However, certain ETEC strains possess one or more enterotoxins but do not contain any known fimbrial or nonfimbrial adhesin. Since adhesin and enterotoxin are key factors that cause diarrhea in piglets, a broad-spectrum vaccine that combines anti-adhesin and anti-enterotoxin would be a highly effective measure for prevention. Therefore, the future direction of vaccine preparations will involve combining heat-resistant and heat-sensitive enterotoxin with major pathogenic adhesin to construct a polyvalent vaccine. This approach aims to prevent piglet diarrhea caused by ETEC through enterotoxin and fimbriae.

Currently, diarrhea caused by ETEC is primarily prevented through drug treatment or immunization using whole-cell vaccines and specific fimbrial vaccines. However, therapeutic and preventive effects of these treatments are not ideal due to the limited effectiveness of the vaccine and the complex and diverse serotypes of pathogenic bacteria. To date, the main preventive measures involve administering the K88–K99 and K88ac-LT_B_ bivalent genetically engineered vaccines. However, the immunogenicity and toxicity issues associated with the main pathogenic factor ST_1_ enterotoxin are not successfully addressed with these vaccines. As a result, the vaccines cannot achieve a satisfactory preventive effect. Therefore, building upon previous research, we mutated the ST_1_ gene to eliminate its natural toxin activity. Since the fimbrial antigen, K99 is also a major virulence factor, we amplified the *K88ac* gene, *K99* gene, *ST*_*1*_ mutant gene, and *LT*_*B*_ gene from the plasmids of *E. coli* C83902 and C83539 using gene mutation, tandem, and fusion techniques. We constructed a recombinant strain BL21(DE3)(pXKK3SL) that contains the *K88ac–K99–3ST*_*1*_ *–LT*_*B*_fusion gene, and the fusion protein was efficiently expressed. The ELISA test confirmed that the fusion protein could bind to ST1 monoclonal antibody, K88ac, K99, and LTB antibodies. Animal experiments showed that the fusion protein had eliminated the natural toxin activity of ST_1_. The immune protection test showed that the inclusion body and inactivated vaccine exhibited a good immune effect. The protection rate of the inclusion body immunization group with 1 MLD challenge dose was 96%, and the protection rate of the inactivated vaccine immunization group with 1 MLD challenge dose was 98%. However, the protection rate of the control group that received the K88–LT_B_ bivalent vaccine was 88%. Therefore, the K88ac–K99–3ST_1_–LT_B_ vaccine we constructed exhibits a positive immune effect. These studies demonstrate that we successfully developed a candidate strain of a tetravalent genetically engineered inactivated vaccine against four major virulence factors of ETEC: K88ac, K99, ST_1_, and LT_B_. This vaccine not only eliminates the natural toxin activity of ST_1_ but also provides immunogenicity. In addition, the inclusion of K88ac and K99 fimbriae further enhances the immune response, preventing diarrhea caused by ETEC through enterotoxin and fimbriae. This study holds significant academic and practical value in effectively controlling diarrhea. In addition, this study establishes a strong foundation for the future development of genetically engineered inactivated vaccines for ETEC, leading to substantial economic and social benefits.

## 5 Conclusions

Animal experiments showed that the tetravalent genetically engineered inactivated vaccine had eliminated the natural toxin activity of ST_1_. Therefore, the disulfide bond of ST_1_ was successfully destroyed by the site-specific mutation technique and the natural toxin activity was lost. The immune protection test demonstrated that the inclusion body and inactivated vaccine exhibited a positive immune effect. The protection rates of the inclusion body group and vaccine group were 96 and 98%, respectively. Therefore, the K88ac–K99–3ST_1_–LT_B_ vaccine generates a highly effective immune response against piglet diarrhea caused by enterotoxin and fimbriae. These results show that the K88ac–K99–3ST_1_–LT_B_ vaccine is a promising candidate for preventing diarrhea in newborn piglets. Overall, although the parenteral immunization strategy is promising, the method is expensive and can lead to stress reactions in piglets due to repeated injections. Previous studies have indicated that multiple antigens in ETEC can potentially be combined. To validate these findings, future studies should be conducted with larger sample sizes, focusing on vaccines that target piglet diarrhea caused by *E. coli*.
